# Positive Reciprocal Regulation of Ubiquitin C-Terminal Hydrolase L1 and β-Catenin/TCF Signaling

**DOI:** 10.1371/journal.pone.0005955

**Published:** 2009-06-18

**Authors:** Anjali Bheda, Wei Yue, Anuradha Gullapalli, Chris Whitehurst, Renshui Liu, Joseph S. Pagano, Julia Shackelford

**Affiliations:** 1 Lineberger Comprehensive Cancer Center, University of North Carolina at Chapel Hill, Chapel Hill, North Carolina, United States of America; 2 Departments of Medicine and Microbiology and Immunology, University of North Carolina at Chapel Hill, Chapel Hill, North Carolina, United States of America; 3 Department of Cell and Developmental Biology, University of North Carolina at Chapel Hill, Chapel Hill, North Carolina, United States of America; Roswell Park Cancer Institute, United States of America

## Abstract

Deubiquitinating enzymes (DUBs) are involved in the regulation of distinct critical cellular processes. Ubiquitin C-terminal Hydrolase L1 (UCH L1) has been linked to several neurological diseases as well as human cancer, but the physiological targets and the regulation of UCH L1 expression *in vivo* have been largely unexplored. Here we demonstrate that UCH L1 up-regulates β-catenin/TCF signaling: UCH L1 forms endogenous complexes with β-catenin, stabilizes it and up-regulates β-catenin/TCF-dependent transcription. We also show that, reciprocally, β-catenin/TCF signaling up-regulates expression of endogenous UCH L1 mRNA and protein. Moreover, using ChIP assay and direct mutagenesis we identify two TCF4-binding sites on the uch l1 promoter that are involved in this regulation. Since the expression and deubiquitinating activity of UCH L1 are required for its own basic promoter activity, we propose that UCH L1 up-regulates its expression by activation of the oncogenic β-catenin/TCF signaling in transformed cells.

## Introduction

The lifetime of many central components of intracellular signaling is regulated by the ubiquitin system [1]. Among them is the multifunctional molecule β-catenin, which plays a dual role in cells as a major structural component of cell–cell adherens junctions and as a signaling molecule in the *Wnt* pathway [2], [3]. As a part of the transcriptional machinery β-catenin provides a transactivation domain in a heterodimeric complex with TCF/Lef transcription factors [4]. β-catenin/TCF/Lef-dependent transcription induces expression of genes such *c-myc*, *cyclin D*, *c-jun*, *survivin* and others, which indicates that β-catenin/TCF/Lef signaling up-regulates oncogenic cellular pathways [5].

The nonjunctional pool of β-catenin is normally a target for destruction by the ubiquitin-proteasome system, and the process of β-catenin regulation through ubiquitination has been studied intensively [6]. The reverse process - deubiquitination–has also been implicated in the regulation of β-catenin intracellular levels [7], and the deubiquitinating enzyme Fam/USP9X was identified as a candidate for β-catenin stabilization [8].

Among the large family of DUBs are Ubiquitin C-terminal Hydrolases–cysteine hydrolases that contain the typical active site triad of cysteine, histidine, and aspartic acid and that catalyze hydrolysis of C-terminal esters and amides of ubiquitin [9]. One of them - UCH L1 - is abundantly (up to 2% of the total soluble protein) expressed in normal brain tissue, and mutations in the UCH L1 gene have been associated with Parkinson's and Alzheimer's diseases [10], [11]. In addition to its deubiquitinating activity, UCH L1 has been shown to exhibit dimerization-dependent ubiquitin ligase activity [12]. Another function of UCH L1 in neurons involves binding and stabilizing mono-ubiquitin *in vivo*, and this function is independent of the enzymatic activity of UCH L1 [13].

There is also growing evidence indicating that UCH L1 is overexpressed in a number of cancers [14], [15], [16], [17], [18], [19], which might be associated with a poor prognosis in some of these cancers. Recent data support this hypothesis, implicating UCH L1 in the up-regulation of metastasis in non-small cell lung cancer [20], and in the proliferation and invasive capacity of malignant B-cells [21]. The possible involvement of UCH L1 in the pathogenesis and progression of human cancer raises the question of how expression of UCH L1 is regulated in transformed cells. The minimal promoter of the *uch l1* gene was cloned and partially characterized in neurons [22], [23], [24], and B-Myb, a transcription factor implicated in the regulation of cell cycle [25], has been shown to stimulate expression of murine *uch l1* on the promoter level *in vitro* and *in vivo* [26], but the regulation of *uch l1* expression in cancer cells is still largely unexplored.

Here we demonstrate a positive feedback between UCH L1 and oncogenic β-catenin/TCF signaling, providing evidence that in transformed cells UCH L1 up-regulates its own expression through β-catenin/TCF-dependent transcription.

## Results and Discussion

**Figure 1:**
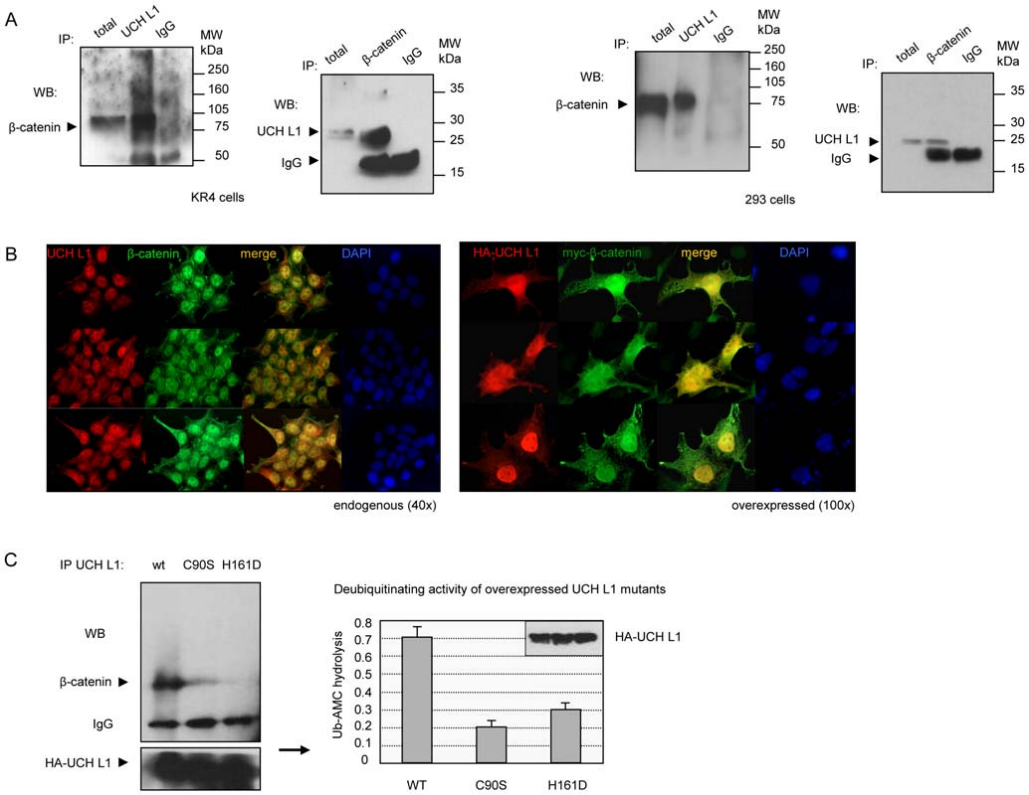
UCH L1 is physically associated with β-catenin. A. Endogenous β-catenin or UCH L1 were immunoprecipitated from KR4 (left) and 293 (right) cells. IPs were resolved in 10–12% PAGE and probed with indicated antibodies. Mouse and rabbit normal immunoglobulins were used as controls for IPs. B. Left panel: nuclear co-localization of UCH L1 and β-catenin. 293 cells were fixed in 4% PFA and double-immunostained with UCH L1 and β-catenin antibodies and red and green fluorescent secondary antibodies. Right panel: 293 cells were transfected with wild type HA-UCH L1 and myc-β-catenin expression vectors. After 24 h cells were fixed and probed with HA and myc antibodies. C. β-catenin is associated with wild type UCH L1, but not with inactive UCH L1 mutants. 293 cells were transfected with wild type and HA-UCH L1 mutants C90S and H161D, and UCH L1 was immunoprecipitated with HA antibody 48 h after transfection. IPs were resolved in 12% PAGE, transferred to PVDF membrane and probed with β-catenin antibody. Enzymatic activity of UCH L1 mutants was verified by *in vitro* hydrolysis of Ub-AMC (right panel).

Previously we have demonstrated that in virus-transformed B-cells β-catenin is physically associated with an active DUB with a molecular weight of ∼26 kDa, and proposed that this DUB is UCH L1 [7], [27]. To verify this suggestion, we immunoprecipitated with specific antibodies endogenous UCH L1 and β-catenin from lymphoid KR4 and epithelial 293 cells. Western blots of IPs (Fig. 1A) demonstrate that β-catenin and UCH L1 form endogenous complexes in cell lines of different origin. Additionally, we performed immunofluorescent co-staining of endogenous and overexpressed β-catenin and UCH L1 in 293 cells (Fig. 1B). UCH L1 and β-catenin were predominantly co-localized in the nucleus, although some cytoplasmic staining for UCH L1 was also observed (Fig. 1B, left).

Similar staining was observed in A-431 carcinoma cell line (http://www.proteinatlas.org/cell_if_unit.php?antibody_id=5993&mainannotation_id=200003070). Co-immunostaining with HA and myc antibodies after co-transfection with HA-UCH L1 and myc-β-catenin expression vectors revealed similar, mostly nuclear co-localization of overexpressed UCH L1 and β-catenin (Fig. 1B, right). Nuclear localization of UCH L1 (PGP9.5) was also observed in lung cancer cell line H1299, where UCH L1 can bind Jab1/Kip1 complexes [28].

The conserved cysteine 90 and histidine 161 in UCH L1 are the necessary catalytic residues for its deubiquitinating activity [9]. We attempted to determine whether the deubiquitinating activity of UCH L1 is important for its ability to form a complex with β-catenin. After overexpression of HA-UCH L1 wild type and mutants C90S and H161D (with cysteine 90 and histidine 161 converted to serine and aspartic acid, respectively [9]), UCH L1 was immunoprecipitated from the cells and the precipitates probed with β-catenin antibody (Fig. 1C, left). Deubiquitinating activity of overexpressed HA-UCH L1 proteins was tested by hydrolysis of the Ub-AMC substrate, which confirmed that enzymatic activity of both C90S and H161D was impaired (Fig. 1C, right). The results indicate that β-catenin is associated preferentially with wild type UCH L1, but not with enzymatically inactive UCH L1 mutants, indicating that the deubiquitinating activity of UCH L1 is required for the molecular events that precede its association with β-catenin.

**Figure 2:**
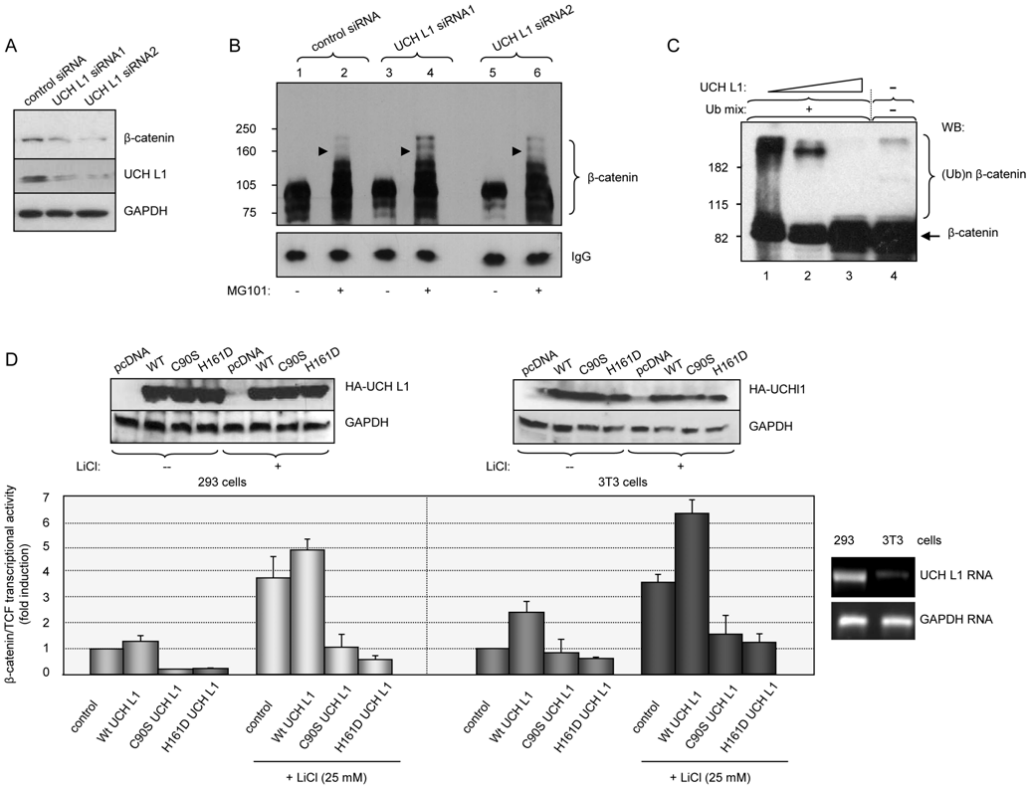
UCH L1 up-regulates β-catenin signaling. A. Reduction of expression of UCH L1 correlates with decrease in β-catenin protein levels. 293 cells were transfected with control GFP siRNA or two UCH L1 siRNAs in pRS vector and maintained in medium containing puromycin. After 3 weeks of selection total lysates from control- and si1/2 UCH L1-transfected cells were resolved in 12% PAGE, transferred to PVDF membrane and probed with β-catenin and UCH L1 antibodies. B. More β-catenin accumulates in UCH L1 siRNA-expressing cells in the presence of proteasome inhibitor. Stable 293 cell lines expressing control and UCH L1 siRNAs were treated for 4 h with 25 mM MG101 or DMSO as negative control. β-catenin was immunoprecicpitated from total lysates, IPs were resolved in 10% PAGE and probed with β-catenin antibodies. Mouse normal immunoglobulins were used as control for IPs. Arrow heads indicate ubiquitinated forms of β-catenin. C. UCH L1 is involved in deubiquitination of β-catenin *in vitro*. β-catenin was *in vitro*-translated (lane 4) as described in Material and Methods. To induce further ubiquitination equal amounts of translated β-catenin were pre-incubated with Ub mix for 30 min, and an *in-vitro* deubiquitination reaction was carried out in the absence (lane 1) or presence of 2 or 4 mM recombinant UCH L1 (lanes 2 and 3). Western blot was performed with β-catenin antibody. D. Deubiquitinating activity of UCH L1 is required for TCF4 transcriptional activity. UCH L1 wild type or UCH L1 inactive mutants C90S and H161D were co-transfected with either TOPFlash or FOPFlash into 293 and 3T3 cells. Luciferase activity and expression of HA-UCH L1 proteins were determined 48 h post-transfection. Where indicated, cells were treated with 25 mM LiCl 6 h before harvesting. The data represent two independent experiments prepared in triplicate and normalized to FOPFlash activity.

To investigate the possible role of UCH L1 expression in the regulation of β-catenin levels, we created 293 cell lines stably expressing two different UCH L1 siRNAs (see Material and Methods). As shown in Fig. 2A, the inhibition of UCH L1 protein expression correlates with reduction of β-catenin levels. To determine whether UCH L1 expression affects β-catenin ubiquitin-dependent proteasomal degradation, we immunoprecipitated endogenous β-catenin from control and UCH L1 siRNA-expressing cells in the presence or absence of the proteasome inhibitor MG101 (Fig. 2B). Western blot analysis demonstrates that while without the inhibitor the amount of β-catenin is reduced in UCH L1 siRNA-expressing cells (compare lanes 3 and 5 to lane 1), the amount of accumulated β-catenin in the presence of MG101 is greater in the cells where UCH L1 expression is inhibited (compare lanes 4 and 6 to lane 2). These results indicate the involvement of UCH L1 in the regulation of β-catenin ubiquitination, and suggest the possibility of direct deubiquitination of β-catenin by UCH L1. To test this possibility, we used *in vitro*-translated and *in vitro*-ubiquitinated β-catenin (Fig. 2C, lanes 4 and 1) as substrate for UCH L1. As shown in Fig. 2C, lanes 2 and 3, the addition of purified UCH L1 results in the disappearance of high molecular weight forms of β-catenin. These results point to β-catenin as a plausible substrate for UCH L1, although they do not eliminate the possibility that UCH L1 effects β-catenin degradation indirectly. For example, deubiquitination of other targets could result in impaired ubiquitination or proteasomal degradation of β-catenin in both *in vivo* and *in vitro* systems. Nevertheless, the observation that β-catenin levels depend on UCH L1 expression raised the question of whether UCH L1 affects β-catenin's function as a transcriptional co-activator.

To examine this possibility, we used luciferase reporter assays to analyze β-catenin/TCF transcriptional activity (Fig. 2D). We utilized HEK 293 and NIH 3T3 cells expressing high and low levels of endogenous UCH L1, which were confirmed by RT-PCR with UCH L1 specific primers (Fig. 2D, right). The cells were co-transfected with reporter plasmids containing binding sites for TCF [29] (see Materials and Methods), along with wild type and enzymatically inactive UCH L1 mutants (expression of UCH L1 proteins was confirmed by western blot with HA antibody, Fig. 2D, top panel). As a positive control for activation of β-catenin-dependent transcription, we used the GSK3β pharmacological inhibitor, LiCl [30]. Overexpression of wild type UCH L1 had very little, if any, effect on the basic or LiCl-induced β-catenin/TCF transcriptional activity in 293 cells (Fig. 2D, left), but in 3T3 cells, the expression of wild type UCH L1 increased β-catenin/TCF transcription significantly (Fig. 2D, right). These results might be explained by different endogenous levels of UCH L1 in 293 and 3T3 cells: so that additional overexpression could not add much to β-catenin/TCF activity in 293 cells, whereas in 3T3 cells which have very low endogenous UCH L1, the effect of exogenous UCH L1 on β-catenin/TCF signaling was more profound. Importantly, expression of the DUB-inactive mutants of UCH L1 C90S and H161D inhibited β-catenin/TCF reporter activity in both cell lines (Fig. 2D), indicating that β-catenin/TCF transcriptional activity depends on the deubiquitinating activity of UCH L1.

Additional information supporting the hypothesis of UCH L1-dependent regulation of β-catenin/TCF signaling was provided from gene expression profiling of stable 293 cell lines expressing control and UCH L1 siRNA: inhibition of UCH L1 reveals reduced expression of several known physiological targets of β-catenin/TCF transcriptional activity such as *c-myc*, *cyclin D1*, *fibronectin* and *stromelysin* (Bheda. A, unpublished data).

The observation that UCH L1 expression is elevated in malignant tumor cells indicates that the *uch l1* gene might be subject to transcriptional regulation during cellular transformation by oncogenic pathways such as β-catenin/TCF. Analysis of the uch l1 promoter using Patch 1.0 has revealed two 5′-TTTGA-3′ putative Lef-1 binding sites on the negative strand [31], pointing to the β-catenin/TCF/Lef complex as a candidate for *uch l1* gene regulation. To examine this possibility, we utilized a reporter construct carrying the luciferase gene under the direction of the 5′ minimal promoter (see Materials and Methods) of the human *uch l1* gene [23].

**Figure 3:**
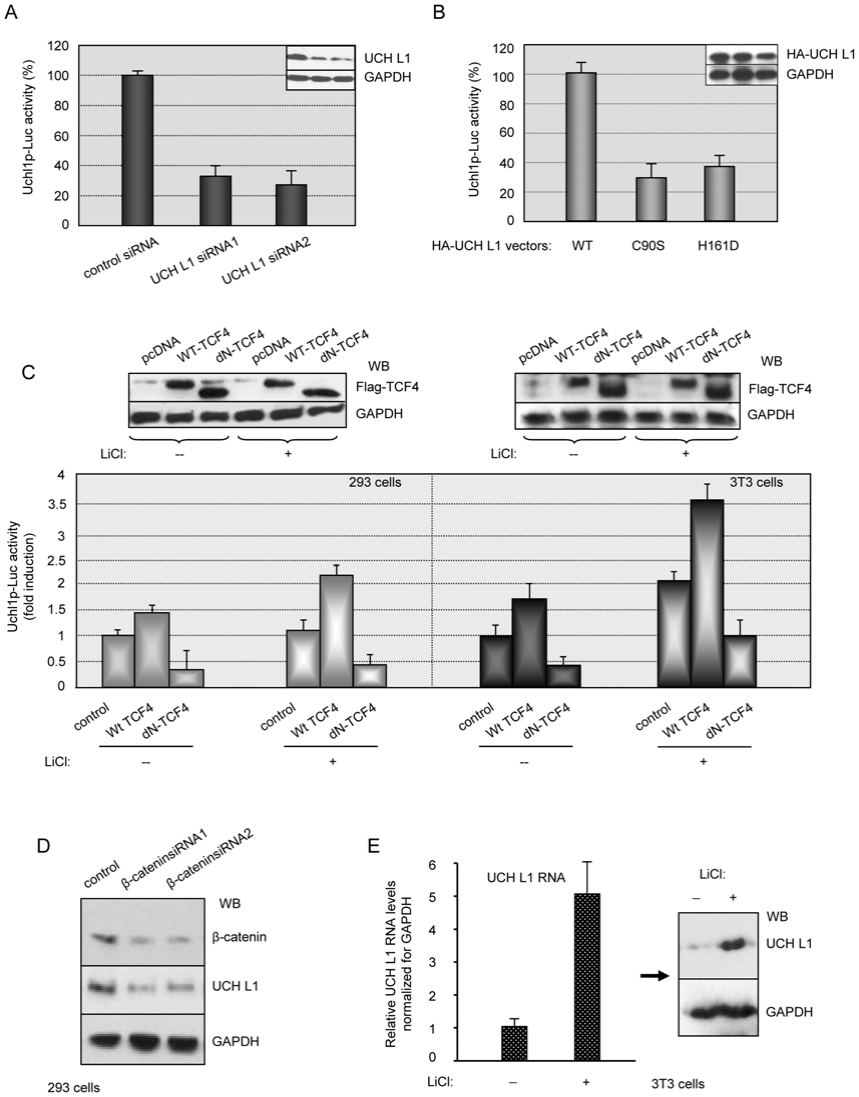
UCH L1 regulates its promoter activity through β-catenin/TCF signaling. A. Expression of UCH L1 is necessary for its promoter activity. Stable 293 cell lines expressing control GFP siRNA and two UCH L1 siRNAs were co-transfected with Uchl1p-Luc reporter plasmid (see Materials and Methods); 48 h post-transfection luciferase activity was measured and normalized to β-gal activity. Reduction in UCH L1 expression was verified by Western blotting. B. Deubiquitinating activity of UCH L1 is required for its promoter activity. Wild type UCH L1 or UCH L1 mutants C90S and H161D expression vectors were transfected into 293 cells along with Uchl1p-Luc reporter. Luciferase assays were performed 24 h post-transfection. The data represent two independent experiments prepared in triplicate and normalized to β-gal activity. C. β-catenin-binding N-terminus of TCF4 is required for TCF4-dependent activation of UCH L1 promoter. Wild type or deltaN TCF4 expression vectors were co-transfected along with Uchl1p-Luc reporter into 293 (left) and 3T3 (right) cells. Luciferase activity and expression of Flag-TCF4 proteins was determined. Where indicated, cells were treated with 25 mM LiCl 6 hour before harvesting. The data represent two independent experiments prepared in triplicate and are normalized for β-gal activity. D. Reduction of β-catenin expression decreases UCH L1 protein levels. 293 cells were transiently transfected with two β-catenin siRNAs in pSUPER.retro vector. Protein levels of β-catenin and UCH L1 were determined by Western blotting 48 h post-transfection. E. Treatment with LiCl induces UCH L1 expression in 3T3 cells. Cells were treated with 25 mM LiCl for 24 h, total RNA isolated, and real-time PCR analysis with specific primers for UCH L1 was performed (left). Total lysates from the same cells were separated in 12% PAGE, transferred to PVDF membrane and probed with UCH L1 antibody (right).

First, we have analyzed uch l1 promoter activity in cell lines stably expressing UCH L1 siRNAs: if our hypothesis about β-catenin/TCF-dependent regulation of *uch l1* is right, then UCH L1 expression should affect its own promoter activity. Indeed, as shown in Fig. 3A, uch l1 promoter (Uchl1p-Luc) activity is significantly lower in cells with a reduced amount of UCH L1. To determine whether UCH L1 deubiquitinating activity is required for its promoter activity we overexpressed wild type and inactive UCH L1 mutants along with the Uchl1p-Luc reporter in control 293 cells (Fig. 3B). Although UCH L1 proteins were expressed at similar levels, uch l1 promoter activity was much lower in the presence of C90S and H161D UCH L1 mutants, indicating that the deubiquitinating activity of UCH L1 is necessary for its promoter activation.

To determine whether β-catenin/TCF transcription up-regulates the uch l1 promoter, we tested Flag-tagged TCF4 wild type and a transcriptionaly inactive N-terminal deletion mutant (dN-TCF4) unable to bind β-catenin [32], for their ability to affect Uchl1p-Luc reporter activity in the presence or absence of the β-catenin activator LiCl in 293 cells (high levels of endogenous UCH L1) and 3T3 cells (low endogenous levels) (Fig. 3C). Overexpression of wild type TCF4 alone had little stimulatory effect on the uch l1 promoter in both cell lines, but wt-TCF4 significantly increased LiCl-dependent activation of uch l1 promoter with a much more profound effect in 3T3 than in 293 cells. Results in Fig. 3C, also demonstrate that the N-terminal deletion mutant of TCF4 inhibited uch l1 promoter activity in both cell lines, even in the presence of LiCl (expression levels of wt-TCF4 and dN-TCF4 were confirmed by western blot (Fig. 3C, top)). Together, these results indicate that TCF4 up-regulates the uch l1 promoter and the effect depends on its ability to bind β-catenin.

To confirm the role of β-catenin signaling in the up-regulation of endogenous UCH L1 expression, we employed two different approaches. First, inhibition of β-catenin expression by transient transfection of β-catenin siRNAs in 293 cells (expressing high levels of UCH L1): Fig. 3D shows that a reduction of β-catenin expression by both siRNAs resulted in decreased levels of endogenous UCH L1 protein. Second, activation of β-catenin signaling in 3T3 cells (express very low levels of UCH L1) by 25 mM LiCl for 24 hours: Fig. 3E demonstrates that the activation of β-catenin with LiCl results in a significant increase of both endogenous UCH L1 RNA (Fig. 3E, left) and protein (Fig. 3E, right) levels.

**Figure 4:**
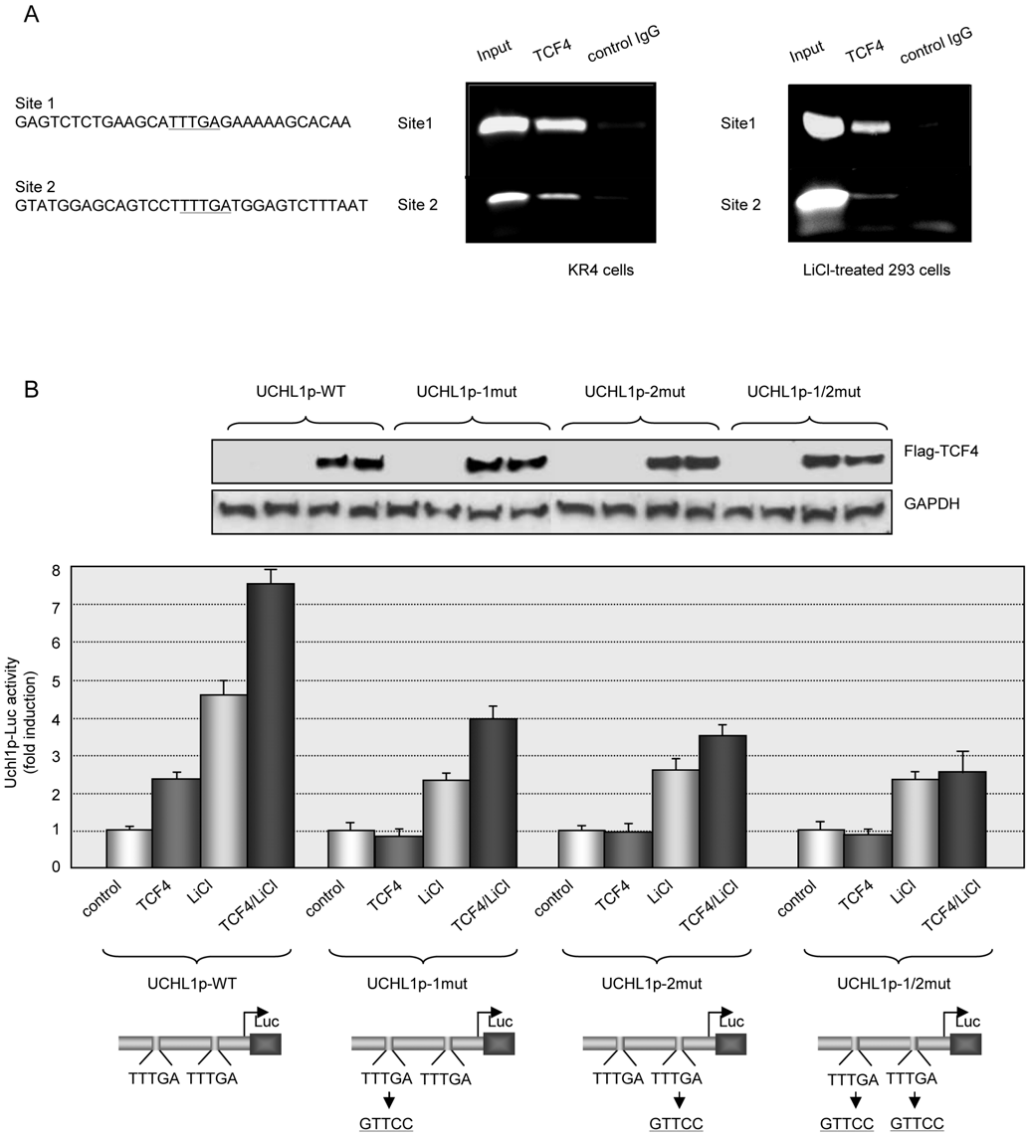
TCF/Lef factors up-regulate UCH L1 expression through direct binding to its promoter. A. TCF4 binds to endogenous uch l1 promoter. ChIP/PCR analysis was performed to determine binding of TCF4 factor to two putative binding sites on uch l1 promoter (left) with the use of specific TCF4 antibody in KR4 or 293 cells treated with 25 mM LiCl. Normal IgGs were used as a negative controls in IPs. PCR reactions were performed with primers targeting TCF4 binding sites on the uch l1 promoter (see Materials and Methods), and amplified DNA products were resolved in 2% agarose gel. B. Mutations in TCF/Lef putative sites inhibit β-catenin/TCF4-dependent activation of the uch l1 promoter. Site-directed mutagenesis of TCF/Lef binding sites was performed as described in Materials and Methods. 3T3 cells were transfected with Uchl1p-Luc wild type and mutant reporter plasmids in the presence or absence of TCF4 expression vector. Luciferase assays were performed 48 h post-transfection and are normalized to β-gal activity. Where indicated 25 mM LiCl was added to the cells 6 h before harvesting. Expression of TCF4 was confirmed by Western blotting.

To determine whether the β-catenin/TCF complex up-regulates UCH L1 expression through direct binding to its promoter, we performed a ChIP assay using the TCF4 antibody followed by DNA isolation and PCR with primers for both putative TCF/Lef sites on the uch l1 promoter in lymphoid KR4 and epithelial 293 cells (Fig. 4A). Non-immunoprecipitated chromatin was used as an input control and normal IgG as a negative control. In lymphoid KR4 cells, TCF4 binding to both putative sites on the uch l1 promoter was detected without additional treatment (Fig. 4A, left); however in 293 cells TCF4-DNA binding was clearly observed after additional activation of β-catenin with LiCl (Fig. 4A, right).

To verify the significance of the putative TCF/Lef binding sites for β-catenin/TCF-dependent activation of the uch l1 promoter, we mutated each of two sites in the promoter, as well as both sites using the wild type uch l1 promoter reporter (UCHL1p-WT) as a template. We transiently transfected wild type and mutant reporters into 3T3 cells and compared their activities (Fig. 4B). To activate β-catenin/TCF signaling, we used co-expression of wild type TCF4, treatment with LiCL, or the combination of both. TCF4 itself was able to activate only UCHL1p-WT, but not any of the three mutant reporters (Fig. 4B, second column in each group). In the presence of LiCl, mutations in each putative TCF/Lef site resulted in a decrease of the promoter activity (Fig. 4B, third column in each group). The combination of TCF4 and LiCl had the strongest cumulative effect on the wild type promoter (UCHL1p-WT). Mutations in each (with a slightly more profound effect on the double-mutant) putative TCF/Lef binding site significantly reduced TCF4/LiCl-dependent activation of Uchl1p-Luc reporters (Fig. 4B, fourth column of each group). Taken together these results identify the uch l1 promoter as a direct target of β-catenin/TCF/Lef transcriptional activity. It is worth noticing, that although mutations in each or both TCF/Lef sites on the uch l1 promoter reduce promoter activity in the presence of LiCl, they do not reduce activity to the basal level. Since LiCl activates β-catenin by inhibition of GSK3β [33], the result suggests that other GSK3β-dependent pathways are involved in the regulation of the uch l1 promoter, in addition to β-catenin/TCF signaling.

While the roles of the multifunctional molecule β-catenin in normal and cancer cells have been studied intensively, the potential impact of UCH L1 in oncogenesis is largely unexplored. Moreover, information on UCH L1 expression in different cancer cell lines is contradictory: on the one hand, silencing of UCH L1 expression by the methylation of its promoter was observed in some cancer cell lines and primary tumors [34], [35], [36]. On the other hand, there is growing evidence indicating that UCH L1 is overexpressed in a number of cancers [14], [15], [16], [17], [18], [19], [37], including recent data demonstrating the invasive capacity of malignant cells conferred by UCH L1 [20], [21]. The observation that expression and enzymatic activity of UCH L1 are required for its basic promoter activity opens speculation that as a deubiquitinating enzyme, UCH L1 might directly or indirectly regulate activity of different transcription factors involved in the regulation of its own promoter. Studies on UCH L1 functions in normal and cancer cells have just begun, and the physiological roles of this molecule in oncogenesis are unknown. Our recent studies demonstrate that proliferation and migration of cell lines stably expressing UCH L1 siRNAs is reduced, and the sensitivity to apoptotic agents was increased [38].

**Figure 5:**
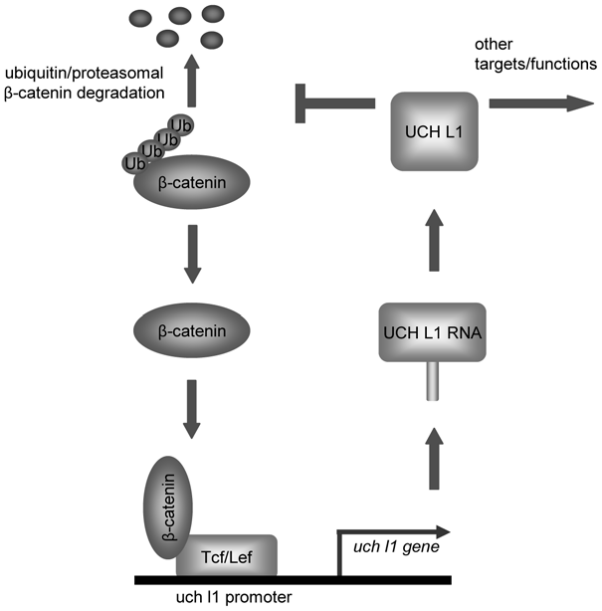
Working hypothesis: UCH L1 up-regulates its expression through β-catenin/TCF signaling. UCH L1 stabilizes β-catenin through deubiquitination and up-regulates β-catenin/TCF transcriptional activity. TCF4/Lef1 transcription factors directly bind to the uch l1 promoter and up-regulate UCH L1 RNA and protein expression.

UCH L1 appears to be a multi-functional protein that might contribute to more diverse cellular processes than were previously thought. Our results indicate that UCH L1 and β-catenin/TCF can positively regulate each other (Fig. 5), supporting the hypothesis that UCH L1 has oncogenic potential, at least under some circumstances.

## Materials and Methods

### Cell Lines and Reagents

Human 293 embryonic kidney cells and NIH 3T3 mouse fibroblast cells were cultured in DMEM (Sigma) supplemented with 10% FBS (Sigma) and penicillin–streptomycin. Lymphoblastoid cell line KR4 [39] were cultured in RPMI 1640 medium plus 10% FBS and penicillin–streptomycin. All cells were maintained at 37°C in 5% CO_2_ in air. MG101, recombinant UCH L1, LiCl and Ubiquitin-AMC were purchased from Calbiochem, Boston Biochem, Sigma and Biomol respectively.

### Expression Plasmids

Wild-type pcDNA3-HA-UCH L1 was a gift from Dr. P. T. Lansbury. pcDNA3-HA-UCH L1 C90S and H161D mutants were generated by inserting a specific mutation at the Cys 90 and H161 sites in wild-type pcDNA3-HA-UCH L1 plasmid with the use of the Quik-Change Site Directed Mutagenesis Kit following the manufacturer's instructions (Stratagene). UCH L1 siRNA was obtained from OriGene Technologies, Inc, in the form of a shRNA expression plasmid. The control siRNA targeted against GFP and was also obtained from OriGene Technologies, Inc. pSuper-retro β-catenin siRNA was constructed as described by the manufactor. TCF reporter plasmids, TOPFlash (optimal TCF-binding site) and FOPFlash (mutated TCF-binding site) were obtained from Upstate Biotechnology. Wild type and deltaN pcDNA3.1-Flag-TCF4 expression vectors were gift from H. Clevers. The UCH L1 promoter region [22] was PCR-amplified from human genomic DNA and cloned into Kpn I and Hind III sites of pGL3 basic plasmid and confirmed by sequencing. The siRNA or primers used in the experiments are as follows:

UCH L1 siRNA–1: 5′TGTGGCACAATCGGACTTATTCACGCAGT 3′

UCH L1 siRNA–2: 5′ CCATGATGCCGTGGCACAGGAAGGCCAAT 3′

β-Catenin siRNA–1: 5′CCATGGAACCAGACAGAAA 3′

β-Catenin siRNA–2: 5′CCCACTAATGTCCAGCGTT 3′

UCH L1 promoter–KpnI: 5′GGTACCAAAACGAACCTCGGTACTGG

UCH L1 promoter–Hind III: 5′AAGCTTCTCACCTCGGGGTTGATCT

### Immunofluorescence staining

293 cells were grown on poly-L-Lysine (Sigma)-coated coverslips in 12-well dishes until 75% confluent. The cells were washed with PBS, fixed with 4% paraformaldehyde for 10 min, permeablized with 0.5% triton, washed three times with PBS and blocked with 5% normal donkey serum. Cells were then incubated with rabbit anti-UCH L1 antibody (Zymed, 1∶100), mouse anti-β-catenin antibody (Zymed, 1∶100), rabbit anti-HA (Santa Cruz, 1∶500), mouse anti-myc (Santa Cruz, 1∶250) in 1∶5 dilution of blocking solution for 1 h at 37°C and then washed three times with PBS. Next, cells were incubated with Alexafluor-594-conjugated goat anti-rabbit and Alexafluor-488-conjugated goat anti-mouse antibodies (Vector Laboratories, 1∶500) for 1 h at 37°C, washed four times with PBS and mounted. Fluorescent images were created using Openlab software (Improvision Inc, MA, USA).

### Immunoblot

Total cell lysates or immunocomplexes were resolved on SDS-PAGE, transferred to nitrocellulose membrane (Osmonics), blocked in 5% milk-Tris-buffered saline solution, and incubated at 4°C overnight with β-catenin (1∶1000, BD Transduction Laboratories, ), UCH L1 (1∶7500, Zymed), GAPDH (1∶5000, Sigma) antibodies. After washing with TBST for 10 min three times, the membrane was incubated with appropriate secondary antibody at room temperature for 1 h, washed three times with TBST as before, treated with SuperSignal (Pierce) detection reagents, and exposed to Kodak XAR-5 film.

### Immunoprecipitation

Cells were lysed with buffer containing of 50 mM, pH 7.6 Tris-HCl, 1% NP-40, 0.25% Na-deoxycholate, 1 mM EDTA, 1 mM Na_3_VO_4_, 1 mM NaF and complete protease inhibitor mixture (Roche Diagnostics). After pre-clearing, cell lysates were incubated with β-catenin (Zymed) or UCH L1 antibody (Zymed) or control Ig G for 1 h in 4°C; immune complexes were then incubated with protein A/G Sepharose beads (Santa Cruz) at 4°C overnight, washed four times with protein lysis buffer, and then eluted from the protein G-Sepharose with 2× Laemmli's buffer by boiling for 3 min.

### *In vitro* Deubiquitination Assays

Endogenous β-catenin was immunoprecipitated from 293 cells treated with 25 µM MG101 for 3 h with specific or control antibodies. Exogenous β-catenin protein was translated in a TNT-coupled reticulocyte-lysate system (Promega) with pCMV β-catenin as template. *In-vitro* translated β-catenin (2 µl) was pre-incubated with 10 mM DTT for 15 min and for 2 h at 30°C in 20 µl of Ub reaction mixture containing: 50 mM Tris-HCl (pH 7.5), 50 mM NaCl, 2 mM Mg-ATP, 20 µM mammalian ubiquitin, 25 µM MG101, 100 nM rabbit E1 and 3 µl reticulocyte lysate, and then incubated with different amounts of UCH L1 recombinant protein (Boston Biochem) The reactions were stopped by adding equal volumes of 2× SDS sample loading buffer followed by Western blot analysis with mouse β-catenin antibody (BD Pharmingen).

### Ubiquitin-AMC assay

Overexpressed HA-UCH L1s were immunoprecipitated with HA antibody from 293 cells transfected with wild type, C90S and H161D uch l1 expression vectors. Immunoprecipitates were incubated with 1 µM of ubiquitin-AMC in reaction buffer containing 50 mM Tris pH 7.5, 2 mM ATP, and 2 mM DTT for 30 min at 37°C. Samples in triplicate were excited by exposure to light at a wavelength of 340 nm, and emission was measured at 460 nm.

### Stable cell lines

For the establishment of UCH L1 siRNA and control siRNA stable lines, 293 cells were transfected with 2 µg of respective plasmid using the Fugene HD (Roche Diagnostics). Cells were passaged 24 h post transfections and selection was started 48 h post transfections. The cells were always maintained in the selective media containing 2 µg/ml puromycin (InvivoGen).

### Transient transfections and Luciferase reporter assay

Cells were transiently transfected with total 2 µg of DNA with Fugene HD reagent (Roche Diagnostics) according to the manufacturer's instructions. Cells were collected 48 h post transfections.

For Luciferase reporter assay, cells were plated in 6-well plates and transiently transfected with the use of Fugene with TCF reporter plasmids, TOPFlash or FOPFlash or Uchl1p-Luc promoter plasmid, and the effector plasmid. The total amount of DNA in all transfection was kept constant with empty vector. Luciferase activities were monitored in cell lysates with the use of Luciferase assay reagents (Promega) 48 h post transfections as described by the manufacturer. All reporter assay results presented here are from three independent experiments prepared in triplicate and have been normalized for β-Gal activity.

### Reverse Transcriptase PCR

HEK 293 and NIH 3T3 cells were treated with 25 mM LiCl for 24 h. Total RNA was extracted with the use of Agilent's Total RNA isolation mini kit (Agilent Technologies). 500 ng of total RNA were used to perform RT-PCR reactions with Qiagen's one step RT-PCR kit (Qiagen) as per manufacturer's instructions at an annealing temperature of 55°C. The samples were analyzed on 1% agarose gel. Primers used:

UCH L1: 5′-GGATGGCCACCTCTATGAAC-3′, 5′-AGACCTTGGCAGCGTCCT-3′, GAPDH: 5′-AGGTGAAGGTCGGAGTCAACG-3′, 5′-AGGGGTCATTGATGGCAACA-3′.

### Quantitative Real Time PCR

NIH 3T3 cells were treated with 25 mM LiCl for 6 h and RNA was isolated. Reverse transcription reaction was performed with 500 ng of total RNA using the iScript cDNA synthesis kit (Bio-Rad). A 1∶25 dilution of cDNA was used in the QRT PCR reaction. QRT-PCR was carried out in a 15 µl reaction mixture with gene-specific primers for UCH L1 and GAPDH (also used in RT-PCR) using iQ-SYBR green kit (Bio-Rad). The PCR conditions were 95°C for 3 min, and 45 cycles of 95°C for 15 s, 55°C for 45 s on the ABI HT 7600 PCR instrument. All samples were assayed in triplicate. The differences in expression of UCH L1 were evaluated using a relative quantification method where the expression of UCH L1 was normalized to the reference gene GAPDH.

### Chromatin Immunoprecipitation

ChIP assay was carried out using Active Motif ChIP-IT enzymatic kit. KR4 and 293 cells were fixed with 37% formaldehyde for 10 min at 37°C, the reaction was stopped with cold 0.125 M Glycine solution for 5 min at RT. The cells were then washed twice with PBS and collected in 0.5 ml of Digestion buffer with protease inhibitors. Chromatin with sheared with shearing enzyme for 10 min at 37°C to obtain an average of 200–1000 bp fragments. Sheared chromatin was incubated overnight at 4°C with Protein G magnetic beads, sheared chromatin, TCF4 antibody (Upstate, Clone 6H53). Immunoprecipitation was performed as per the manufacturer's instructions, cross-linking was reversed by incubated the immunoprecipitated complexes with 5 M NaCL for 2 h at 65°C followed by Proteinase-K treatment for 2 h at 42°C. PCR reaction was performed with 5 µl precipitated DNA using the primer pairs flanking consensus TCF sites in UCH L1 promoter. PCR conditions: one cycle, 95°C for 2 min; 30 cycles of 95°C for 30 s, 55°C for 30 s, and 72°C for 2 m, and a final extension at 72°C for 10 min. The primers used in the reaction are: Site 1 (5′ CATTTACATTCATTCGTATT 3′, 5′ CCTTTCACCATCCCAATTAC 3′) and Site 2 (5′ ATGGGTTTCCAGAAACTTCG 3′, 5′ TGGTTGTGGAGACGGGATTT 3′).

### Site Directed Mutagenesis

The UCH L1 promoter was mutated at different TCF4/Lef1 binding sites using the Quik-Change Site Directed Mutagenesis Kit (Stratagene). The TCF4/Lef1 binding site *“TTTGA”* was mutated to *“CCTTG”* and exactly same mutations were introduced at both the binding sites. Four nucleotide changes were introduced into the parental plasmid by oligonucleotide primers carrying the specific mutations. The promoter was mutated at a single TCF4/Lef1 binding site or both binding sites at a time. The parental DNA template was then digested with *Dpn*I. All mutations and the integrity of the remainder of the promoter were confirmed by DNA sequencing.
